# Characteristics of health-state utilities used in cost-effectiveness analyses: a systematic review of published studies in Asia

**DOI:** 10.1186/s12955-023-02131-z

**Published:** 2023-06-20

**Authors:** Zhihao Yang, Xueyun Zeng, Weidong Huang, Qingqing Chai, Angela Zhao, Ling-Hsiang Chuang, Bin Wu, Nan Luo

**Affiliations:** 1 Health Services Management Department, Guizhou Medical University, Gui’an, China; 2 Center of Medicine Economics and Management Research, Guizhou Medical University, Gui’an, China; 3 Shanghai Chest Hospital, Shanghai Jiao Tong University School of Medicine, Shanghai, China; 4 School of Health Management, Harbin Medical University, Harbin, China; 5 Department of Pharmacy, School of Medicine, Huangpu Branch, Shanghai Ninth People’s Hospital, Shanghai Jiao Tong University, Shanghai, China; 6 Analysis Group, Boston, MA USA; 7 Department of Epidemiology and Global Health, Umeå University, Umeå, Sweden; 8 GongJing Healthcare (Nanjing) Co. Ltd, Nanjing, China; 9 School of Medicine, Ren Ji Hospital, Shanghai Jiao Tong University, Shanghai, China; 10 Saw Swee Hock School of Public Health, National University of Singapore, Singapore, Singapore

**Keywords:** Cost-effectiveness analysis, Health-state utility, Asia, QALY

## Abstract

**Introduction:**

Cost-utility analysis (CUA) is the preferred form of economic evaluation in many countries. As one of the key data inputs in cost-utility models, health state utility (HSU) has a crucial impact on CUA results. In the past decades, health technology assessment has been expanding rapidly in Asia, yet research examining the methodology and process used to generate cost-effectiveness evidence is scarce. The aim of this study was to examine the reporting of the characteristics of HSU data used in CUAs in Asia and how the characteristics have changed over time.

**Methods:**

A systematic literature search was performed to identify published CUA studies targeting Asian populations. Information was extracted for both the general characteristics of selected studies and the characteristics of reported HSU data. For each HSU value identified, we extracted data for four key characteristics, including 1) estimation method; 2) source of health-related quality of life (HRQoL) data; 3) source of preference data; and 4) sample size. The percentage of nonreporting was calculated and compared over two time periods (1990–2010 vs 2011–2020).

**Results:**

A total of 789 studies were included and 4,052 HSUs were identified. Of these HSUs, 3,351 (82.7%) were from published literature and 656 (16.2%) were from unpublished empirical data. Overall, the characteristics of HSU data were not reported in more than 80% of the studies. Of HSUs whose characteristics were reported, most of them were estimated using the EQ-5D (55.7%), Asian HRQoL data (91.9%), and Asian health preferences (87.7%); 45.7% of the HSUs was estimated with a sample of 100 or more individuals. All four characteristics showed improvements after 2010.

**Conclusion:**

Over the past two decades, there has been a significant increase in CUA studies targeting Asian populations. However, HSU’s characteristics were not reported in most of the CUA studies, making it difficult to evaluate the quality and appropriateness of the HSUs used in those cost-effectiveness studies.

**Supplementary Information:**

The online version contains supplementary material available at 10.1186/s12955-023-02131-z.

## Background

Economic evaluations are widely utilized to guide healthcare decision-making, and in many countries, cost-utility analysis (CUA) is the preferred form of economic evaluations [1]. CUA estimates health gains in terms of quality-adjusted life-years (QALYs), a measure combining life expectancy and quality of life, quantified using health-state utilities (HSUs) [2]. HSU is a measure of the value of health outcomes, or health-related quality of life (HRQoL), usually based on the stated preferences of the concerned general public. In current practice, HSU is measured on a cardinal scale, where 1 represents full health, 0 represents being dead, and negative values indicate states worse than dead [3]. There are different ways of obtaining HSU data including use of preference-based measures (PBM), direct elicitation techniques, and mapping algorithms [4].

HSU is a crucial data input in cost-utility models, and it significantly impacts the results of cost-utility analyses (CUA) [3–5]. However, there is no universally accepted method for determining HSU values, and different approaches may yield varying HSU values for the same health states [6, 7]. These variations can lead to different CUA conclusions which may undermine the goal of consistent decision-making [7, 8]. As a result, some health technology assessment (HTA) agencies have recommended methods for deriving HSU data in their technical guidance. For instance, England's National Institute for Health and Care Excellence (NICE) recommends using EQ-5D and its value set, based on the health preferences of the general UK population [9]. Nevertheless, there is no international consensus on the best practices for generating and utilizing HSUs in CUA [1]. Furthermore, the reporting of HSU information in CUA models is often inadequate [5, 7, 10, 11]. Frequently, studies do not reference the original sources, and did not report essential details such as preference sources, sample sizes, elicitation techniques, and justifications for the selected utility values [5, 7, 10, 11].

In the past few decades, HTA has been rapidly developing in Asia [12]. To address the increasing healthcare demands and costs, some Asian countries have formally adopted HTA [12, 13]. For instance, South Korea introduced the positive list system in 2006 and established a formal HTA process to inform reimbursement decisions for new drugs [14]. Other national HTA agencies in Asia include the Health Intervention & Technology Assessment Program (HITAP) in Thailand, the HTA Section within the Ministry of Health in Malaysia, and the Agency for Care Effectiveness (ACE) in Singapore [15]. Despite the rapid development, many challenges still persist, including a lack of well-trained personnel, tools, and local data, which might have impeded the quality of the evidence generated [16, 17].

In spite of the HTA growth in Asia, there is a dearth of research examining the methodology and process for cost-effectiveness evidence generation. Only one study investigated the methodological quality of CUA studies targeting Asian populations. In a systematic review of 175 CUA studies published between 2000 and 2012, Thorat et al. found that good methodology was generally adhered to, although some reporting issues were present [15]. However, this study did not assess the quality of HSUs, which plays a vital role in CUA and decision-making. In this study, we aimed to systematically evaluate the HSU data quality in published CUA studies targeting Asian populations by examining 1) the proportion of studies which failed to report the details of HSU data used; and 2) the reported characteristics of the HSU data including estimation method, preference source, HRQoL data source, and sample size of reported HSU data. We also examined how the reporting and characteristics of HSU evolved over the past few decades. We hope that findings in this review can facilitate the improvement of the practices related to HSU reporting and usage in economic evaluations in Asia.

## Methods

### Data sources and search strategies

A systematic literature search was performed to identify published CUA studies targeting Asian populations, which is defined as populations of any Asian ethnicity living in Asia. Four databases, including PubMed, Web of Science, Medline, and Embase, were systematically searched from inception to December 2019. The searches used the following search terms: “cost-utility analysis”, “cost–benefit analysis”, “cost-effectiveness analysis”, “economic evaluation”, “QALY” and “Asia”. The search algorithms can be found in the Additional file 1.

### Inclusion/Exclusion criteria

Studies were included if they: 1) were economic evaluations; 2) used QALY as the outcome measure and reported the use of HSU; 3) targeted an Asian population; and 4) were available in English. Studies were excluded if they were reviews, editorials, conference abstracts, or methodological articles, or if their full text was not available.

### Data extraction

Two authors (XZ and QC/AZ) independently selected studies and extracted data using a standardized Microsoft Excel-based data extraction form. Any disagreement was resolved by consulting the corresponding author (NL).

Information was extracted for both the general characteristics of selected studies and the characteristics of reported HSU data. Extracted study characteristics included year of publication, studied population/country, type of intervention, study perspective, time frame of the analysis, model type, type of sensitivity analysis, and discount rate. We also reviewed the included studies to understand whether any literature or systematic reviews was undertaken to obtain HSU values.

Extraction of the HSU data characteristics was restricted to the HSU data used in the base-case analysis of each study. For each CUA, we first identified all the health states used in the estimation of QALYs. Next, we identified the HSUs for the respective health states used in the base-case analysis. As a result, the number of HSUs we extracted was equal to the number of health states used in the CUA studies. For each HSU value identified, we extracted data for four key characteristics, including 1) estimation method, e.g., PBMs, direct elicitation methods, mapping etc.; 2) source of health-related quality of life (HRQoL) data, being defined as the country, district or region from which HRQoL data was collected and used to estimate the HSU values; 3) source of preference data, being defined as the country, district or region from which health preferences were elicited and used to estimate the HSU values; and 4) sample size, being defined the population sample size used for collecting i) HRQoL data (in the case of using an indirect estimation method, such as EQ-5D) or ii) health preference data (in the case of using a direct estimation methods such as time trade-off).

### Data analysis

All the four key HSU characteristics were coded into categorical variables and analyzed at the HSU level. First, for each HSU characteristic, we calculated the percentage of nonreporting, that is, the proportion of HSUs for which the characteristic was not reported. We also examined how the proportion changed from years 1999–2010 to years 2011–2019. To examine the changes in reporting quality, we additionally conducted sub-group analysis for the top three countries for which most CUA studies were conducted. To investigate changes in HSU estimation methods, we excluded HSUs estimated using expert opinions or unjustifiable methods, and categorized the remaining HSUs into one of three groups including EQ-5D, SF-6D/HUI/QWB/mapping, and time trade-off (TTO)/standard gamble (SG)/visual analogue scale (VAS). For sources of HRQoL and preference data, we identified for each HSU whether the sources matched the country targeted by the CUA, and calculated the proportion of HSUs estimated using HRQoL and preference data from the country which the CUA was conducted for.

## Results

### Study selection

**Fig. 1:**
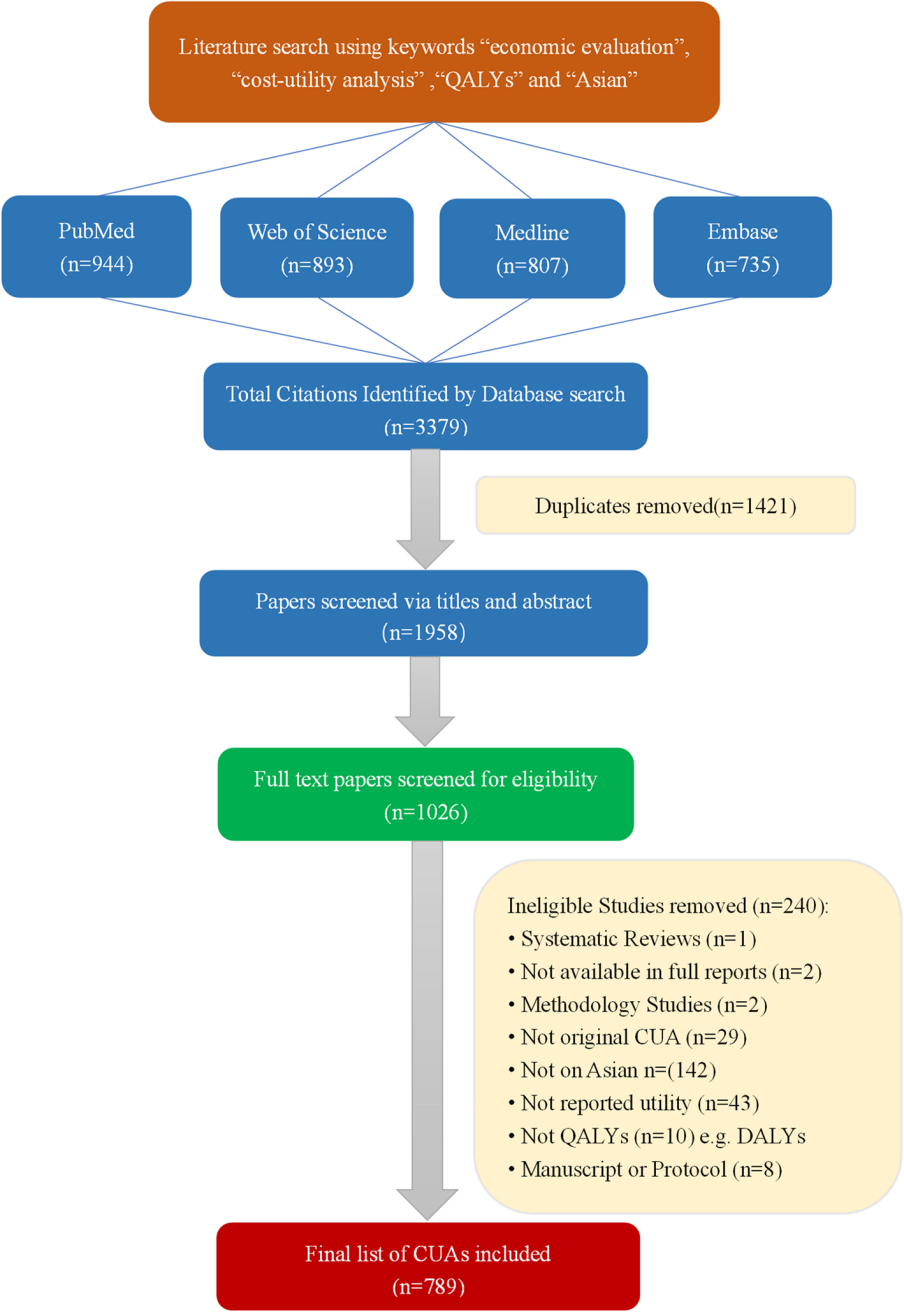
Flow diagram showing the process of study selection. QALYs, quality-adjusted life-years; CUA, cost-utility analysis; DALYs, disability-adjusted life years

The study selection process is summarized in Fig. 1. The initial search identified a total of 3,379 studies from four databases. After removing duplicates, 1,958 studies were included in the review process. Based on assessment of titles and abstracts, 932 studies were excluded. Of the remaining 1026 studies, full-text review excluded 237 studies. Among those, 142 did not target an Asian population; 53 did not use QALY as the outcome measure (*n* = 10) or report any HSU data (*n* = 43); 29 were not original CUAs; 11 were protocols or unpublished manuscripts (*n* = 8), methodological articles (*n* = 2), or systematic review (*n* = 1); and 2 were not available in full-text. Therefore, a total of 789 studies were included. Among those, 577 studies obtained HSUs from published literature and 123 studies used unpublished empirical data to calculate the HSUs.

### Study characteristics

**Fig. 2:**
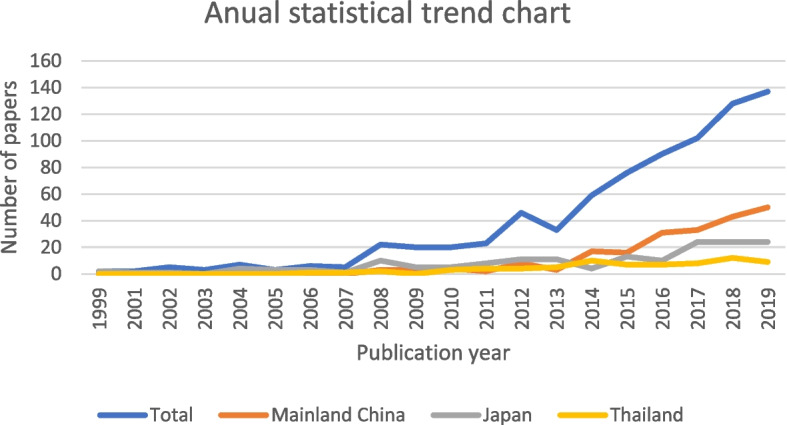
The number of published QALY-based CUA studies in Asia by year

The studies included in this review were published between 1999 and 2019, with the majority published in the period 2017 to 2019 (43.9%) or 2014 to 2016 (26.6%). There was a significant increase in the number of studies after 2013 (Fig. 2). Most of the CUA studies targeted the population of mainland China (27.0%), Japan (20.8%), or Thailand (9.3%). The majority of the CUAs evaluated a therapeutic intervention (73.0%), took a payer’s perspective (54.0%), and used a time horizon of more than 10 years (52.0%). The CUA studies were primarily model-based, using either Markov models (53.0%) or decision tree models (12.5%), and a probabilistic sensitivity analysis (62.2%). Detailed characteristics of the included studies are summarized in Table 1. Notably, only 5% of the CUA studies reported the use of a literature review to identify HSU values.

**Table 1:** Characteristics of included papers (*n* = 789)

Characteristic	Category	Number of papers	Percentage
			(%)
Targeting population	China (mainland)	213	27
	Japan	164	20.8
	Thailand	73	9.3
	Korea	58	7.4
	Hong Kong	56	7.1
	Taiwan	54	6.8
	Singapore	46	5.8
	Iran	34	4.3
	India	20	2.5
	Malaysia	18	2.3
	Others^a^	53	6.7
Type of intervention	Screening or diagnosis	102	12.9
	Prevention (e.g. vaccination/public intervention)	96	12.2
	Therapy (e.g. surgery/pharmaceutical)	576	73
	Disease management	14	1.8
	Other (diabetes pay-for-performance program)	1	0.1
Study perspective	Patient	14	1.8
	Provider/payer	426	54
	Societal	249	31.6
	Not reported	100	12.7
Time horizon	Less than 1 year	27	3.4
	1–5 years	112	14.2
	6-10 years	62	7.9
	More than 10 years	410	52
	Not reported	178	22.6
Type of model	Markov models	418	53
	Decision tree models	99	12.5
	Both	85	10.8
	Other	70	8.9
Type of sensitivity analysis	None	69	8.7
	Non-probabilistic one/multi way sensitivity analysis	229	29.1
	Probabilistic sensitivity analysis	491	62.2
Discount rate used for cost	None	177	22.4
	1%-2%	32	4
	3%	446	56.5
	3.5%-7.2%	134	17
Discount rate used for outcome	None	177	22.4
	1%-2%	34	4.3
	3%	450	57
	3.5%-7.2%	128	16.2
Identification of HSUs	Systematic review	19	2.4
	Non-systematic review	21	2.7
	Not reported	749	94.9

*HSU* Health state utility

^a^Including following countries: Israel (10); Turkey (8); Vietnam (6); Mixed countries (6); Indonesia (5); Saudi Arabia (4); Philippines (3); Jordan (2); Kazakhstan (2); Lebanon (2); Bhutan (1); Cambodia (1); Oman (1); Sri Lanka (1); The United Arab Emirates (1)

### Characteristics of HSUs

A total of 4,052 HSUs were identified from the base-case analyses of the included studies. The number of HSUs per study ranged from 1 to 25, with the median being 5. Of the 4,052 HSUs, 3,351 (82.7%) were from published literature and 656 (16.2%) were unpublished empirical data. The full characteristics of the HSUs are shown in Table 2.

**Table 2:** Characteristics of HSUs included in the review (*n* = 4,052)

Characteristic	Category	n	% (of all HSUs)	% (of HSUs without missing data in the characteristic)
Estimation method	EQ-5D	781	19.3	55.7
	SF-6D	147	3.6	10.5
	HUI	54	1.3	3.9
	QWB	5	0.1	0.4
	TTO	170	4.2	12.1
	SG	87	2.1	6.2
	VAS	38	0.9	2.7
	Mapping	67	1.7	4.8
	Expert opinion	32	0.8	2.3
	Unjustifiable methods	20	0.5	1.4
	Not reported/missing	2651	65.4	-
Country of sample	China (mainland)	133	3.3	14.2
	Japan	242	6.0	25.8
	Thailand	104	2.6	11.1
	South Korea	92	2.3	9.8
	Iran	51	1.3	5.4
	Taiwan	46	1.1	4.9
	Singapore	67	1.7	7.1
	Hong Kong	47	1.2	5.0
	Malaysia	28	0.7	3.0
	India	19	0.5	2.0
	Other Asian countries^a^	33	0.8	3.5
	Non-Asian countries^b^	76	1.9	8.1
	Not reported/missing	3114	76.9	-
Sample size	n < 50	200	4.9	31.4
	50 ≤ n < 100	146	3.6	22.9
	n ≥ 100	291	7.2	45.7
	Not reported/missing	3415	84.3	-
Country of preferences	China (mainland)	46	1.1	12.6
	Japan	72	1.8	19.7
	Thailand	48	1.2	13.2
	Hong Kong	27	0.7	7.4
	South Korea	39	1.0	10.7
	Singapore	21	0.5	5.8
	Malaysia	16	0.4	4.4
	Taiwan	28	0.7	7.7
	Other Asian countries^c^	23	0.6	6.3
	Non-Asian countries^d^	45	1.1	12.3
	Not reported/missing	3687	91.0	-

*HSU* Health state utility, *HRQoL* Health-related quality of life, *HUI* Health Utilities Index, *QWB* Quality of Well-Being, *TTO* Time trade-off, *SG* Standard gamble, *VAS* Visual analogue scale

^a^Including following countries: Indonesia (10); Israel (6); Lebanon (6); Saudi Arabia (5); Vietnam (4); Turkey (2)

^b^Including following countries: UK (34); US (24); Sweden (9); Australia (1)

^c^Including following countries: Iran (12); India (6); Sri Lanka (2)

^d^Including following countries: UK (26); US (11); Australia (6); Germany (2)

### Nonreporting

**Fig. 3:**
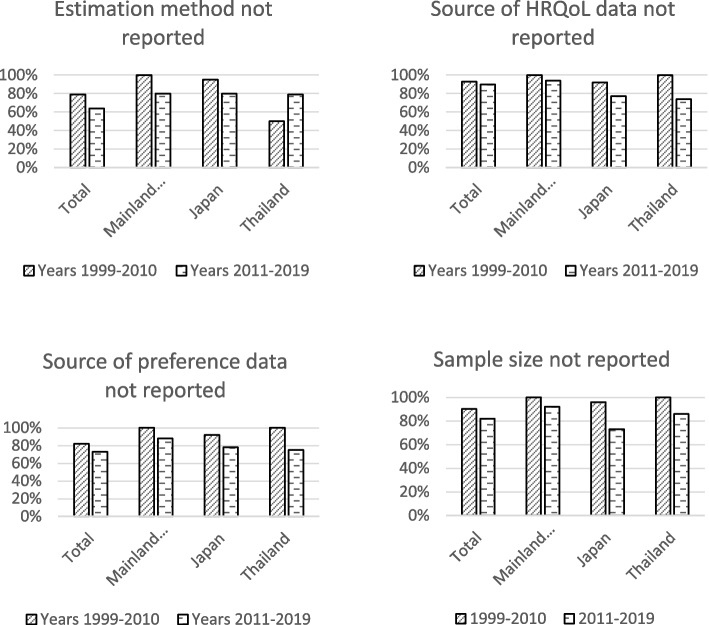
The proportion of HSUs whose characteristics were not reported by publication year

Key characteristics were not reported for the majority the 4,052 HSUs. The percentage of nonreporting was 65.4% for estimation methods, 76.9% for source of sample, 84.3% for sample size, and 91.0% for source of health preferences. Figure 3 shows that the percentages of nonreporting decreased after year 2010 as a whole and for three countries with the most publications, except that Thailand had a higher percentage of estimation method nonreporting after year 2010.

### Estimation method

**Fig. 4:**
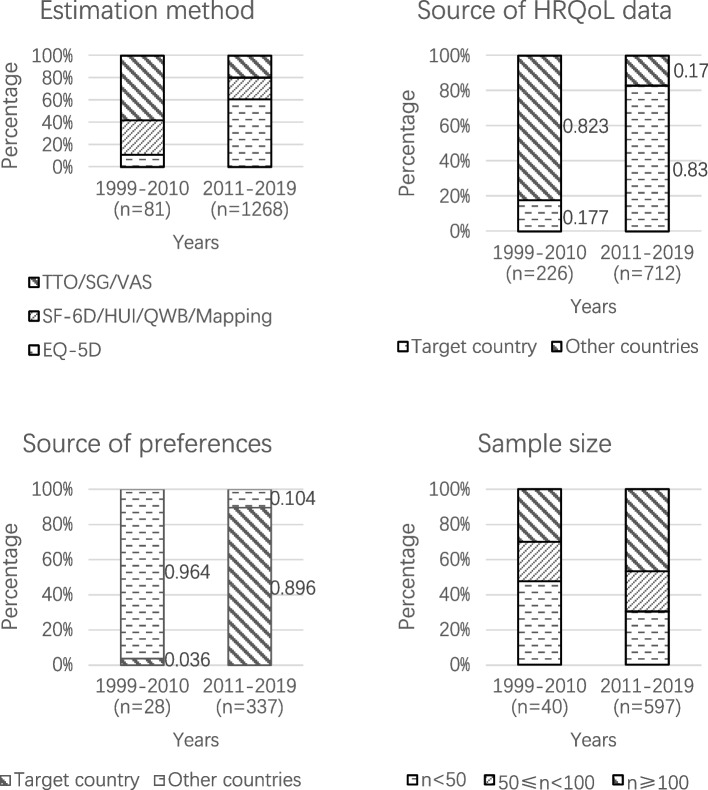
Characteristics of HSUs by publication year

Of 1,349 HSUs for which estimation methods were reported, EQ-5D (*n* = 781, 55.7%) was the most frequently used method, followed by TTO (*n* = 170, 12.1%) and SF-6D (*n* = 147, 10.5%). A total of 20 HSUs (0.5%) were obtained using an unjustifiable method, for example, using non-utility HRQoL instrument. Figure 4 shows that there was a significant increase in the use of EQ-5D after 2010.

### Source of HRQoL data

In total, the HRQoL data source was reported for 938 HSUs. Most of the HSU (*n* = 862, 91.9%) were estimated using Asian HRQoL data (see Table 2), with Japan (25.8%), mainland China (14.2%), and Thailand (11.1%) being the main source. The majority of the HSUs (*n* = 631, 67.3%) were estimated using HRQoL data collected from the target country and the proportion of such HSUs increased considerably after 2010 (Fig. 4).

### Source of preference data

The source of preferences was reported for only 365 of the 4,025 HSUs. Among those, 320 HSUs (87.7%) were derived from an Asian value set (see Table 2). The majority of the health preferences used were from Japan (19.7%), Thailand (13.2%), mainland China (12.6%), or a non-Asian country (12.3%). Most HSU values (*n* = 303, 83.0%) were estimated using health preference data collected from the target country, with a significant increase of such HSUs after 2010 (Fig. 4).

### Sample size

Among the 637 HSUs for which the estimation sample size was reported, almost half of these values was estimated with a sample of 100 or more individuals (45.7%) and the proportion increased after 2010 (Fig. 4).

## Discussion

In this study, we systematically summarized the HSU characteristics in published CUA studies targeting Asian populations. There is an evident increase in CUA publications in the past two decades in Asia, especially after the year 2013. This upward trend in the number of CUA studies is not surprising because countries in this region have been actively using economic evaluations to inform reimbursement decision-making within their respective health systems in the past two decades [13, 18–20].

Despite the proliferation in publications, most studies failed to report the basic characteristics of the HSU values used. Although the issue of nonreporting showed an improvement, the estimation method, source of HRQoL data, source of preference data, and sample size were not reported for over 60% to 90% of the HSUs used in the CUA studies published between years 2011–2019. This finding is in line with previous studies of CUA studies not targeting a specific region. For example, Ara et al. found that out of 24 published CUA studies in the cardiovascular disease area published after 2014, none of the studies reported HSU-related details (e.g., sample size, estimation method) used [7]. These findings suggest that poor reporting quality for HSUs used in CUA studies may be a global issue and not just specific to Asia. This suboptimal practice is disappointing since ISPOR’s Consolidated Health Economic Evaluation Reporting Standards (CHEERS) [21] was published in 2013 simultaneously in ten international health economics and medical journals. The targets of this reporting guidance are researchers as well as editors and reviewers of journals publishing economic evaluation studies, including CUA. CHEERS recommends economic evaluation studies to describe HSU estimation methods, together with the population sample size, and sources of the HRQoL and preference data [21]. It is worth noting that those recommendations have been maintained in CHEERS-2022 [22], which was simultaneously published in twenty-one journals. Hopefully, this updated guidance will help improve the HSU data reporting quality in future economic evaluation studies.

It is concerning that a very large proportion of CUA studies used HSUs from the literature but did not report how those HSUs were identified. It is possible that the HSUs were identified through comprehensive literature reviews but the CUA papers failed to report them. If so, this is a reporting issue that should be improved in future studies [22]. If the HSUs in those CUA studies were not identified using formal literature reviews, the appropriateness and validity of the HSUs as well as the conclusions of those CUAs would be questionable. Future CUA studies in Asia should adopt a more rigorous approach in searching, evaluating, and selecting HSUs by following emerging good practices such as those recommended by an ISPOR taskforce [10].

Among the HSUs for which basic characteristics were reported, the majority of those used in CUA studies published after 2010 were derived using recommended methodology such as using an established preference-based measure EQ-5D and its value sets reflecting the health preferences of targeted populations. The trend of using EQ-5D to acquire HSU data is consistent with findings from CUA-related systematic reviews worldwide [23–26]. For example, in a systematic review of HSU values for stroke, 87 of 111 (78%) studies used EQ-5D to generate HSU data. The predominant use of EQ-5D data in CUAs around the world is probably attributable to both recommendations of HTA agencies [13] and its extensive adoption by outcome researchers and clinicians. A recent review study found that there is a proliferation of clinical studies using EQ-5D as an outcome measure [27], forming a database that provides CUA studies with HSU data. In Asia, most HTA agencies recommend the use of either EQ-5D or validated preference-based measures, and local public’s health preferences to estimate HSU values [13]. Also importantly, many Asian countries have established their national EQ-5D value sets [28–30] and validated the desirable measurement properties of EQ-5D in their populations [31].

A main limitation of this study is that we only included CUA studies published in English. We explored the possibility of including CUA studies published in both English and non-English such as Chinese literature into this review. We found it is technically difficult and resource-demanding, and eventually decided to only include English databases in this study. Nevertheless, in a similar systematic review study evaluating QALY-based CUA studies in the Chinese literature, we found similar trends [32]. Another important limitation of this review is the poor reporting quality of the reviewed studies. Only a small portion of the reviewed CUA studies reported the characteristics of the HSUs used. As a result, our findings may not reflect the true status quo of Asian CUA studies. This is also because there are many unpublished CUA studies. In HTA practice, CUA studies may be performed with limited resources and are therefore of low quality. For example, Hong and Bae found that, in addition to poor reporting, CUA reports submitted to the Health Insurance Review and Assessment Service (HIRA) in South Korea between 2014 and 2018 inadequately adhered to the recommendation of using HSUs reflecting Korean’s health preferences [33]. Lastly, we did not investigate the extent to which HSUs in the literature were appropriately used in the CUAs. A previous review of CUA studies in cardiovascular disease identified potentially inappropriate use of published HSU data such as modifying original HSU values without justification [19].

## Conclusion

Over the past two decades, the number of CUA studies targeting Asian populations significantly increased and the methods used to derive HSUs in those studies improved. However, HSU characteristics were not reported in most of published Asian CUA studies, making it difficult to evaluate the true quality and appropriateness of the HSUs used in those cost-effectiveness studies. We advocate better reporting of HSUs in future CUAs.

## Supplementary Information

**Additional file 1.**

## Data Availability

Upon request to the corresponding author.
