# Supplementary material for: Characteristics of health-state utilities used in cost-effectiveness analyses: a systematic review of published studies in Asia
Source: Health Qual Life Outcomes. 2023 Jun 20;21:59. doi: 10.1186/s12955-023-02131-z (PMC10283288; doi:10.1186/s12955-023-02131-z)
Supplement: Supplementary file 1 — Additional file 1. [file 12955_2023_2131_MOESM1_ESM.docx]

**PubMed**

(((((((((((((((((("Analysis, Cost-Benefit"[Title/Abstract] OR "Cost-Benefit Analyses"[Title/Abstract]) OR "Cost Benefit Analysis"[Title/Abstract]) OR "Analysis, Cost Benefit"[Title/Abstract]) OR "Cost Benefit Analyses"[Title/Abstract]) OR "Cost Effectiveness"[Title/Abstract]) OR "Effectiveness, Cost"[Title/Abstract]) OR "Cost-Utility Analysis"[Title/Abstract]) OR "Analysis, Cost-Utility"[Title/Abstract]) OR "Cost Utility Analysis"[Title/Abstract]) OR "Cost-Utility Analyses"[Title/Abstract]) OR "Economic Evaluation"[Title/Abstract]) OR "Economic Evaluations"[Title/Abstract]) OR "Cost Benefit"[Title/Abstract]) OR "Costs and Benefits"[Title/Abstract]) OR "Benefits and Costs"[Title/Abstract]) OR "Cost-Effectiveness Analysis"[Title/Abstract]) OR "Analysis, Cost-Effectiveness"[Title/Abstract] AND (((((("Quality-Adjusted Life Year"[Title/Abstract] OR "Quality Adjusted Life Years"[Title/Abstract]) OR "QALY"[Title/Abstract]) OR "Healthy Years Equivalents"[Title/Abstract]) OR "Healthy Years Equivalent"[Title/Abstract]) OR "Adjusted Life Years"[Title/Abstract]) OR "Adjusted Life Year"[Title/Abstract]) NOT "DALY"[Title/Abstract]) NOT "disability-adjusted life year"[Title/Abstract] NOT "DALYs"[Title/Abstract] NOT "disability-adjusted life-years"[Title/Abstract]) AND (("china"[MeSH Terms] OR "china"[All Fields]) OR ("asian continental ancestry group"[MeSH Terms] OR ("asian"[All Fields] AND "continental"[All Fields] AND "ancestry"[All Fields] AND "group"[All Fields]) OR "asian continental ancestry group"[All Fields] OR "chinese"[All Fields]) OR ("taiwan"[MeSH Terms] OR "taiwan"[All Fields]) OR ("hong kong"[MeSH Terms] OR "hong kong"[All Fields]) OR ("turkey"[MeSH Terms] OR "turkey"[All Fields]) OR ("korea"[MeSH Terms] OR "korea"[All Fields]) OR ("asian continental ancestry group"[MeSH Terms] OR ("asian"[All Fields] AND "continental"[All Fields] AND "ancestry"[All Fields] AND "group"[All Fields]) OR "asian continental ancestry group"[All Fields] OR "korean"[All Fields]) OR ("japan"[MeSH Terms] OR "japan"[All Fields]) OR ("asian continental ancestry group"[MeSH Terms] OR ("asian"[All Fields] AND "continental"[All Fields] AND "ancestry"[All Fields] AND "group"[All Fields]) OR "asian continental ancestry group"[All Fields] OR "japanese"[All Fields]) OR ("bangladesh"[MeSH Terms] OR "bangladesh"[All Fields]) OR ("singapore"[MeSH Terms] OR "singapore"[All Fields]) OR ("thailand"[MeSH Terms] OR "thailand"[All Fields]) OR ("asian continental ancestry group"[MeSH Terms] OR ("asian"[All Fields] AND "continental"[All Fields] AND "ancestry"[All Fields] AND "group"[All Fields]) OR "asian continental ancestry group"[All Fields] OR "thai"[All Fields]) OR ("india"[MeSH Terms] OR "india"[All Fields]) OR ("vietnam"[MeSH Terms] OR "vietnam"[All Fields]) OR ("asian continental ancestry group"[MeSH Terms] OR ("asian"[All Fields] AND "continental"[All Fields] AND "ancestry"[All Fields] AND "group"[All Fields]) OR "asian continental ancestry group"[All Fields] OR "vietnamese"[All Fields]) OR ("laos"[MeSH Terms] OR "laos"[All Fields]) OR ("cambodia"[MeSH Terms] OR "cambodia"[All Fields]) OR ("asian continental ancestry group"[MeSH Terms] OR ("asian"[All Fields] AND "continental"[All Fields] AND "ancestry"[All Fields] AND "group"[All Fields]) OR "asian continental ancestry group"[All Fields] OR "cambodian"[All Fields]) OR ("indonesia"[MeSH Terms] OR "indonesia"[All Fields]) OR ("philippines"[MeSH Terms] OR "philippines"[All Fields]) OR ("malaysia"[MeSH Terms] OR "malaysia"[All Fields]) OR ("bhutan"[MeSH Terms] OR "bhutan"[All Fields]) OR ("pakistan"[MeSH Terms] OR "pakistan"[All Fields]) OR ("sri lanka"[MeSH Terms] OR "sri lanka"[All Fields]) OR ("kazakhstan"[MeSH Terms] OR "kazakhstan"[All Fields]) OR ("iran"[MeSH Terms] OR "iran"[All Fields]) OR ("jordan"[MeSH Terms] OR "jordan"[All Fields]) OR ("lebanon"[MeSH Terms] OR "lebanon"[All Fields]) OR ("israel"[MeSH Terms] OR "israel"[All Fields]) OR ("saudi arabia"[MeSH Terms] OR "saudi arabia"[All Fields]) OR ("oman"[MeSH Terms] OR "oman"[All Fields]) OR ("georgia"[MeSH Terms] OR "georgia"[All Fields] OR "georgia (republic)"[MeSH Terms] OR "georgia (republic)"[All Fields]) OR ("armenia"[MeSH Terms] OR "armenia"[All Fields]) OR ("cyprus"[MeSH Terms] OR "cyprus"[All Fields])) AND ((Clinical Trial[ptyp] OR Journal Article[ptyp]) AND "loattrfull text"[sb] AND English[lang]) NOT systematic review[Title/Abstract] NOT review[Title/Abstract] NOT meta-analysis[Title/Abstract]

**Web of Science**

((TS= (("economic evaluation" OR "cost utility analy*" OR "cost benefit analy*" OR "cost effectiveness analy*" OR "Analysis, Cost-Benefit" OR "Cost-Benefit Analyses" OR "Analysis, Cost Benefit" OR "Cost Effectiveness" OR "Effectiveness, Cost" OR "Cost-Benefit Data" OR "Cost Benefit Data" OR "Cost-Utility Analysis" OR "Analysis, Cost-Utility" OR "Cost-Utility Analyses" OR "Economic Evaluations" OR "Cost Benefit" OR "Costs and Benefits" OR "Benefits and Costs" OR "Cost-Effectiveness Analysis" OR "Analysis, Cost-Effectiveness") AND ("QALYs" OR "quality-adjusted" OR "quality-adjusted life year" "Quality Adjusted Life Years" OR "Adjusted Life Years" OR "Adjusted Life Year")) AND CU= ("China" OR “Chinese” OR "Taiwan" OR "Hong Kong" OR "Turkey" OR "Korea" OR “Korean” OR "Japan" OR “Japanese” OR "Bangladesh" OR "Singapore" OR "Thailand" OR "India" OR" Vietnam" OR “Vietnamese” OR "Laos" OR "Cambodia" OR “Cambodian” OR "Indonesia" OR "Philippine" OR "Malaysia" OR "Bhutan" OR "Pakistan" OR "Sri Lanka" OR "Kazakhstan" OR "Iran" OR "Jordan" OR "Lebanon" OR "Israel" OR "Saudi Arabia" OR "United Arab Emirates" OR "Oman" OR "Georgia" OR "Armenia" OR "Cyprus") NOT ALL=( "DALY" OR “DALYs” OR "disability-adjusted life year" OR “ disability-adjusted life-years” OR “systematic review” OR “systematic” OR “review” OR “meta-analysis”))) AND Language: (English) AND Article types: (Article)

**MEDLINE**

((("QALYs" or "quality-adjusted" or "quality-adjusted life year" or "Quality Adjusted Life Years" or "Adjusted Life Years" or "Adjusted Life Year") not "DALY") and ("economic evaluation" or "cost utility analy*" or "cost benefit analy*" or "cost effectiveness analy*" or "Analysis, Cost-Benefit" or "Cost-Benefit Analyses" or "Analysis, Cost Benefit" or "Cost Effectiveness" or "Effectiveness, Cost" or "Cost-Benefit Data" or "Cost Benefit Data" or "Cost-Utility Analysis" or "Analysis, Cost-Utility" or "Cost-Utility Analyses" or "Economic Evaluations" or "Cost Benefit" or "Costs and Benefits" or "Benefits and Costs" or "Cost-Effectiveness Analysis" or "Analysis, Cost-Effectiveness") and (China or Chinese or Taiwan or Hong Kong or Turkey or Korea or Korean or Japan or Japanese or Bangladesh or Singapore or Thailand or Thai or India or Vietnam or Vietnamese or Laos or Cambodia or Cambodian or Indonesia or Philippine or Malaysia or Bhutan or Pakistan or Sri Lanka or Kazakhstan or Iran or Jordan or Lebanon or Israel or Saudi Arabia or United Arab Emirates or Oman or Georgia or Armenia or Cyprus))

**EMBASE**

((('economic evaluation' OR 'cost utility analy*' OR 'cost benefit analy*' OR 'cost effectiveness analy*' OR 'analysis, cost-benefit' OR 'cost-benefit analyses' OR 'analysis, cost benefit' OR 'cost effectiveness' OR 'effectiveness, cost' OR 'cost-benefit data' OR 'cost benefit data' OR 'cost-utility analysis' OR 'analysis, cost-utility' OR 'cost-utility analyses' OR 'economic evaluations' OR 'cost benefit' OR costs) AND benefits OR benefits) AND costs OR 'cost-effectiveness analysis' OR 'analysis, cost-effectiveness') AND ('qalys' OR 'quality-adjusted' OR 'quality-adjusted life year' OR 'quality adjusted life years' OR 'adjusted life years' OR 'adjusted life year') AND (china:ab,ti OR chinese:ab,ti OR taiwan:ab,ti OR 'hong kong':ab,ti OR turkey:ab,ti OR korea:ab,ti OR korean:ab,ti OR japan:ab,ti OR japanese:ab,ti OR bangladesh:ab,ti OR singapore:ab,ti OR thailand:ab,ti OR thai:ab,ti OR india:ab,ti OR vietnam:ab,ti OR vietnamese:ab,ti OR laos:ab,ti OR cambodia:ab,ti OR cambodian:ab,ti OR indonesia:ab,ti OR philippine:ab,ti OR malaysia:ab,ti OR bhutan:ab,ti OR pakistan:ab,ti OR 'sri lanka':ab,ti OR kazakhstan:ab,ti OR iran:ab,ti OR jordan:ab,ti OR lebanon:ab,ti OR israel:ab,ti OR 'saudi arabia':ab,ti OR 'united arab emirates':ab,ti OR oman:ab,ti OR georgia:ab,ti OR armenia:ab,ti OR cyprus:ab,ti) NOT ('daly':ab,ti OR 'dalys':ab,ti OR 'disability-adjusted life year':ab,ti OR 'disability-adjusted life-years':ab,ti OR 'systematic review':ab,ti OR 'systematic':ab,ti OR 'review':ab,ti OR 'meta-analysis':ab,ti) AND [article]/lim AND [english]/lim
